# Protective Factors Modulate the Risk of Beta Amyloid in Alzheimer's Disease

**DOI:** 10.1155/2020/7029642

**Published:** 2020-10-29

**Authors:** Bin Zhou, Kenichiro Tanabe, Shinsuke Kojima, Satoshi Teramukai, Masanori Fukushima, The Alzheimer's Disease Neuroimaging Initiative

**Affiliations:** ^1^Translational Research Center for Medical Innovation, Foundation for Biomedical Research and Innovation, Kobe, Japan; ^2^Department of Biostatistics, Kyoto Prefectural University of Medicine, Kyoto, Japan

## Abstract

**Aim:**

To identify the factors protecting Abeta-positive subjects with normal cognition (NC) or mild cognitive impairment (MCI) from conversion to Alzheimer's disease (AD).

**Methods:**

Subjects with MCI in the Alzheimer's Disease Neuroimaging Initiative (ADNI) database, with baseline data for neuropsychological tests, brain beta amyloid (Abeta), magnetic resonance imaging (MRI), APOE genotyping, and 18F-FDG-PET (FDG), were included for analysis.

**Results:**

Elevated brain amyloid was associated with a higher risk of conversion from MCI to AD (41.5%) relative to Abeta levels of <1.231 (5.5%) but was not associated with conversion from NC to AD (0.0 vs. 1.4%). In the multivariate Cox regression analyses, elevated Abeta increased the risk of AD, while higher whole-brain cerebral glucose metabolism (CGM) assessed by FDG decreased the risk of AD in subjects with the same amount of Abeta. Even in the patients with heavily elevated brain amyloid, those with FDG > 5.946 had a lower risk of AD. ApoE4 carrier status did not influence the protective effect.

**Conclusion:**

Higher average CGM based on FDG modified the progression to AD, indicating a protective function. The results suggest that the inclusion of this CGM measured by FDG would enrich clinical trial design and that increasing CGM along with the use of anti-Abeta agents might be a potential prevention strategy for AD.

## 1. Introduction

Alzheimer's disease (AD) is a progressive neurodegenerative disease that lacks an effective treatment. The pathogenesis proceeds for decades before the onset of symptoms. Current research shows that the annual conversion rate from mild cognitive impairment (MCI) to AD is approximately 10.2 to 33.6% based on a systematic review by Ward et al. [1]. Many factors associated with the conversion from MCI to AD have been explored. Elevated beta amyloid (Abeta) levels in the brain or cerebrospinal fluid (CSF) increase the risk of converting from normal cognition (NC) or MCI to AD [2–4]. Currently, a positive amyloid status, apolipoprotein E4 (ApoE4) carrier status, and elevated brain Abeta are risk factors for AD [5–7] and are inclusion criteria in clinical trial designs [8–10]. Subjective cognitive complaints (SCC) may inform the risk for future cognitive decline and track progression of self-perceived decline, particularly in those along the AD trajectory [10]. However, one study also suggested that elevated Abeta alone may be insufficient to produce cognitive change in individuals at risk for AD and supports the use of multiple biomarkers to stage AD progression [5, 11]. Some subjects with a positive amyloid status remained cognitively normal during long-term follow-up [5].

Obviously, some of the factors that mediate the synthesis of amyloid in the pathogenesis of AD and the factors that decrease the risk are not yet clear. Arterial spin labelling magnetic resonance imaging (ASL-MRI) and 18F-fluorodeoxyglucose positron emission tomography (FDG) have comparable diagnostic accuracy in AD [12]. Changes in the cerebral blood flow (CBF) and brain FDG occur in different brain regions in Abeta-positive subjects across the AD continuum compared with Abeta-negative NC subjects; however, another study suggested the changes may have been the result of methodological differences [13]. In patients with MCI, FDG demonstrated hypometabolism and a component in the precuneus [14]. Hypoperfusion in the right precuneus, inferior parietal lobule, and middle cingulate gyrus were associated with the conversion from MCI to AD [15]. Many studies have shown that reductions in FDG were associated with conversion to AD, and the combination of FDG scanning with measurement of hippocampal volume resulted in 96% specificity and 92% sensitivity in the prediction of conversion [16, 17]. However, other studies indicated that a positive FDG scan in people with MCI was of no clinical value in the early prediction of progression to AD dementia [18, 19]. Decreased ASL values, mainly in the parietal and frontotemporal areas, were shown in a group of patients with the first signs of cognitive instability [20]. Whether these changes in CBF and uptake of FDG, which are physiological factors, can modulate the progression to AD in Abeta-positive subjects is not yet clear.

We used the ADNI data, including the pathophysiological factors of brain Abeta measured by PET, brain structure by MRI, and cerebral glucose metabolism (CGM) by FDG to explore the factors that mediate the conversion to AD in Abeta-positive and Abeta-negative MCI and NC subjects. We did not include neuropsychological tests, to avoid circularity issues in prediction.

## 2. Subjects and Methods

### 2.1. Subjects

We included all subjects with MCI or NC in the ADNI 1 and ADNI GO/2 phases obtained from the publicly available data repository, the Alzheimer's Disease Neuroimaging Initiative (ADNI) database (adni.loni.usc.edu).

The primary goal of ADNI has been to test whether serial MRI, PET, other biological markers, and clinical and neuropsychological assessments can be combined to measure the progression of MCI and early AD.

The data used in this article were downloaded from the ADNI website in May 2019, including ADNIMERGE and updated diagnosis information. The data included florbetapir-PET scans to obtain a measure of cerebral amyloidosis, FDG, UCSF structural MRI, APOE genotyping, and some cognitive tests. For up-to-date information on specific inclusion and exclusion criteria, please see http://adni.loni.usc.edu.

### 2.2. Psychometric Testing

The following measure was included in the analysis: mini-mental state examination (MMSE); we did not include other neuropsychological tests to avoid circularity issues related to the use of these tests for diagnosis.

### 2.3. Determination of Brain Amyloid Status by PET

Florbetapir-PET was conducted in accordance with the ADNI PET protocols, which are available online (http://adni.loni.usc.edu/data-samples/pet). Image processing was performed by the ADNI core laboratory as described by Landau et al. [2]. A PET scan was acquired 50–70 minutes after injection of florbetapir. Images were smoothed and aligned to an MPRAGE anatomic image to obtain cortical segmentation. A mean cortical standardized uptake value ratio (SUVr) in the lateral and medial frontal, anterior and posterior cingulate, lateral parietal, and lateral temporal regions was obtained. A cortical florbetapir uptake value of ≥1.11 was considered “elevated” or “positive” for cortical Abeta [2].

### 2.4. FDG Measures

The methods used to assess CGM based on 18F-FDG-PET have been described online (http://adni.loni.usc.edu/methods/pet-analysis-method/pet-analysis/). Further details on the quality control analyses and procedures to enhance uniformity and reduce variability in PET images across centers are provided by Joshi et al. [21]. Quantitative CGM maps were intensity normalized to average brainstem FDG uptake. The metaregion of interest (meta-ROI) included is the mean SUVr of the temporal, angular, and posterior cingulate gyri [22].

MRI was performed using standardized protocols on 1.5 T MRI scanners with 3D T1-weighted sequences optimized for the different scanners, as indicated by http://www.loni.ucla.edu/ADNI/Research/ [7]. All images were corrected for spatial distortion due to gradient nonlinearity and normalized for B1 nonuniformity (see also http://www.loni.ucla.edu/ADNI/Data/). MRI measurements were reconstructed with the software program FreeSurfer, as described in detail by Fischl et al. [7]. The average cortical thickness (TA), standard deviation of thickness (TS), surface area (SA), and cortical volume (CV) were calculated. The automated Hippocampus Multi-Atlas Propagation and Segmentation (HMAPS) method was also used to measure several structures, such as the hippocampal volume. The factors included the CV, TA, and SA of the following regions: the left and right entorhinal cortices, left and right hippocampi, left and right inferior temporal lobes, left and right parahippocampi, and left and right superior temporal lobes. Measurements from the left and right olfactory lobes, left and right hippocampi, left and right parahippocampi, medial portion of the orbital frontal cortices, lateral portion of the occipital cortex, inferior temporal gyrus, temporal pole, and the isthmus of the cingulate cortex were averaged to form a meta-ROI thought to be sensitive to early AD-related neurodegeneration.

APOE genotyping was performed as described on the ADNI website (http://adni.loni.usc.edu/data-samples/genetic-data/).

### 2.5. Statistics

Two-tailed Student's *t*-tests with equal variances and Welch's *t*-test for unequal variance were used to assess differences in demographic characteristics between Abeta-positive and Abeta-negative subjects. Wilcoxon's rank sum test was used only for follow-up time. Fisher's exact test was used for category variables.

Cox proportional hazard regression models were used to identify the statistically significant variables used to explore the risk factors for AD. For variable selection in multivariate Cox regression analysis, the backward elimination method was used with the removal criterion *P* value ≥ 0.1. Similarly, in the survival tree analyses, the entry criterion was *P* value < 0.15. The multiple testing problem was not considered. The cut-off points of Abeta and FDG tests were generated by the survival tree at the first cut-off value.

Statistical analyses were performed using SAS version 9.4 (SAS Institute, Inc. Cary, NC, USA) and R version 3.6.1.

## 3. Results

Baseline brain PET amyloid data were available for 424 MCI and 265 NC subjects. Of these, 188 (44.3%) of the 424 MCI subjects were Abeta positive while 52 (19.6%) of the 265 NC subjects were Abeta positive. The mean age of the Abeta-negative group was younger than that of the Abeta-positive group in both the MCI and NC subjects. Age and ApoE status were significantly different between the Abeta-negative and Abeta-positive groups in both the MCI and NC subjects. The median follow-up period was 25.87 (range: 4.72–60.89), 32.76 (range: 5.54–60.07), 19.47 (range: 5.51–47.67), and 22.13 (range: 5.05–48.46) months from baseline in the Abeta-positive and Abeta-negative subjects in the MCI and NC groups, respectively (Table 1).

### 3.1. Cut-Off Values Determined by Survival Tree Method

We compared several methods based on multivariate Cox regression analysis (median, ROC curve, and survival tree) to find the optimal cut-off values for Abeta and FDG. These were determined to be 1.231 and 5.946, respectively, by the survival tree method at the first point.

In the subjects with Abeta values greater than 1.231, a further cut-off point was found at 1.462 where those with Abeta values > 1.462 had a higher risk of conversion to AD compared with those with Abeta values between 1.231 and 1.462 (Suppl. Figure 1).

### 3.2. Outcomes of MCI and NC Subjects Based on Brain Abeta

**Figure 1:**
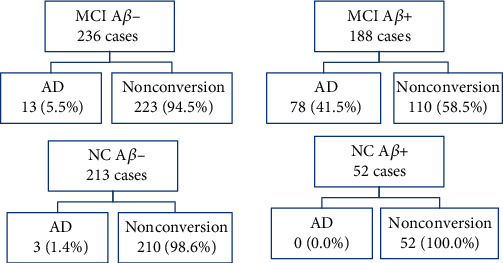
Outcomes of MCI and NC subjects based on PET brain Abeta. AD: Alzheimer's disease; NC: normal cognition; MCI: mild cognitive impairment; A*β*- and A*β*+: brain PET imaging Abeta negative and Abeta positive, respectively.

Of the 424 MCI subjects, 91 converted to AD. Although those who were Abeta positive had a higher risk of converting to AD (78, 41.5%) than those who were Abeta negative, the majority of Abeta-positive MCI subjects (110, 58.5%) had not converted over a median period of 25.87 months of follow-up. If we assume that all subjects who were Abeta positive could develop AD during long-term follow-up, this difference in speed of conversion should be noted. Of the NC subjects, the 3 who converted to AD came from the group of 213 Abeta-negative NC subjects, and there was no conversion to AD in the 52 Abeta-positive NC subjects (Figure 1). The survival curves showed that at the proper cut-off points, Abeta status is a better predictor of disease progression than FDG (not shown) (Suppl. Figure 2).

### 3.3. Risk and Protective Factors Associated with Conversion from MCI or NC to AD

We found a significant difference in higher average brain FDG values between the conversion and nonconversion groups in both Abeta-positive and Abeta-negative subjects. However, the factors intracranial volume, fusiform gyrus volume, thickness of the entorhinal cortex, and hippocampal volume were significantly different in the conversion and nonconversion groups only in the Abeta-positive subjects (Suppl. Table 1). The results of the univariate and multivariate Cox regression analyses showed that higher average brain FDG, thickness of the entorhinal cortex, and hippocampal volume were protective factors for AD, while higher brain Abeta and ApoeE4 carrier status increased the risk of conversion to AD (Table 2).

### 3.4. Risk Classification by Brain PET Abeta and FDG

**Figure 2:**
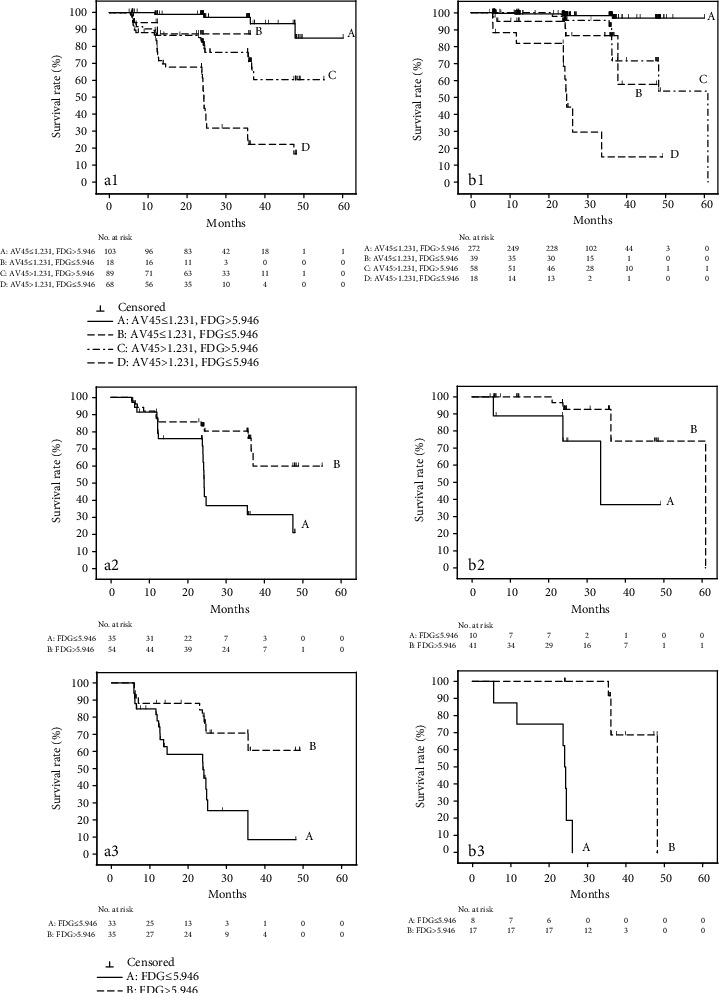
Risk classification by the combination of FDG with brain PET Abeta. (a) Survival curves by combination of Abeta and FDG in ApoE4 carriers. (a1, A) Abeta ≤ 1.231 and FDG > 5.946; (B) Abeta ≤ 1.231 and FDG ≤ 5.946; (C) Abeta > 1.231 and FDG > 5.946; and (D) Abeta > 1.231 and FDG ≤ 5.946. (a2, A) 1.231 < Abeta ≤ 1.462 and FDG ≤ 5.946 and (B) 1.231 < Abeta ≤ 1.462 and FDG > 5.946. (a3, A) 1.462 < Abeta and FDG ≤ 5.946 and (B) 1.462 < Abeta and FDG > 5.946. (b) Survival curves by combination of Abeta and FDG in non-ApoE4 carriers. (b1, A) Abeta ≤ 1.231 and FDG > 5.946; (B) Abeta ≤ 1.231 and FDG ≤ 5.946; (C) Abeta > 1.231 and FDG > 5.946; and (D) Abeta > 1.231 and FDG ≤ 5.946. (b2, A) 1.231 < Abeta ≤ 1.462 and FDG ≤ 5.946 and (B) 1.231 < Abeta ≤ 1.462 and FDG > 5.946. (b3, A) 1.462 < Abeta and FDG ≤ 5.946 and (B) 1.462 < Abeta and FDG > 5.946.

Risk classification using the combination of brain Abeta and FDG indicated in the Cox regression analysis showed that the lowest risk group consisted of those subjects with Abeta ≤ 1.231 and FDG > 5.946, and the subjects with the highest risk of conversion to AD were those with Abeta > 1.231 and FDG ≤ 5.946. Even in the group with very highly elevated Abeta, greater than 1.462, the protective effect of higher FDG still remained; there was a large difference in the conversion rate for the higher and lower FDG groups. Following stratification by ApoE4 status, higher FDG still showed a protective effect in the different groups. In ApoE4 noncarriers, the hazard ratio for Abeta > 1.231 and FDG ≤ 5.946 was much higher than in ApoE4 carriers (Table 3 and Figure 2).

## 4. Discussion

Elevated brain Abeta indicated a higher risk of conversion from MCI to AD; however, in the subjects with elevated brain Abeta, approximately 60% did not develop AD during the follow-up. The factors that mediated Abeta pathogenesis were ApoE4 and/or other factors. Our analysis found that increased CGM measured by FDG was associated with delayed progression to AD in both the Abeta-positive and negative subjects, indicating a protective function. Even in the subjects with heavy Abeta deposition, the protective effect still remained and was independent of the ApoE4 carrier status.

Cerebral glucose hypometabolism and low CBF have been reported in MCI and AD patients [12, 16–18]. Alterations in FDG have been observed at least 20 years before positive Abeta measurements. Subtle cognitive dysfunction has been observed at least 10 years before patients test positive for Abeta [23]. In familial AD, the cascade of Abeta, then altered metabolism and then atrophy is definitive [24]. Khosravi et al.'s study revealed FDG global quantification to be a superior indicator of cognitive performance in the AD and MCI patients compared to 18F-florbetapir-PET [25]. However, recent research has indicated that increased Abeta deposition leads to the progression to mild cognitive impairment but decreased glucose metabolism does not contribute to progression [26, 27]. Not all subjects with elevated Abeta in the brain show a decrease in CGM. Our analysis indicated that even in some subjects with heavily elevated Abeta, the CGM can still be high and that higher CGM may modulate the risk of Abeta causing progression to AD, rather than being a diagnostic indicator.

Many papers have discussed unsuccessful trials in the AD field over the last 20 years [28–30] that focused on incorrect therapeutic targets and had methodological problems in the clinical trial design or began treatment too late. In these studies, the endpoint is a critical issue for clinical trial design [28]. However, we know that the inclusion criteria are also important because discrepancies between subjects can no doubt influence assessment of the efficacy of the interventions in these clinical trials. MCI subjects selected based on current technology are heterogeneous because their outcomes are very different, as shown in our analysis; this may have contributed to the failure of prevention and treatment in clinical trials. Both diagnosis and recruitment of appropriate trial participants are challenging. Targeting subjects with high risk can reduce the sample size, but the reduction in variability across subjects could facilitate the efficacy assessment. Many studies have proposed methods to select subjects at high risk [31–33]; some of the proposed risk factors have included decreased cognition, measured by the Clinical Dementia Rating Scale Sum of Boxes (CDR-SOB), Alzheimer's Disease Assessment Scale-cognitive subscale (ADAS-cog), and auditory verbal learning test (AVLT), atrophy of the hippocampus and entorhinal cortex, and elevated brain Abeta and tau. Poor cognitive performance clearly implies disease progression. One of the limitations of these models is that the circularity of including neuropsychological tests in the diagnosis might mask the true risk and protective factors. Our analysis showed that the factors associated with pathophysiological processes were able to classify the risk of transition to AD very well in subjects positive for brain Abeta. Measurement of CGM by FDG combined with brain Abeta is suggested for the enrichment of clinical trial designs.

CGM as measured by FDG has been closely connected to neuronal activity. CGM can be used as a marker of synaptic dysfunction before an advance in neurodegeneration. In subjects with elevated brain Abeta, the altered metabolism measured by FDG was observed to reduce the progression of AD. One hypothesis is that we can delay AD progression by increasing CGM.

In a previous study, six months of cognitive training increased CGM only in the MCI patients; however, there was no significant association between increased FDG uptake and improved cognition [34]. Physical activity has also been shown to alter brain glucose metabolism; however, these studies appear to be limited to conditions of high-intensity exercise in young and middle-aged cognitively normal cohorts. Aerobic training improved cognition and changed cerebral glucose metabolism in areas related to cognition in subjects with MCI [35]. Increased FDG uptake can also be due to an increase in CBF. Exercise training altered CBF and improved cognitive performance in older adults with and without cognitive impairment [36] and altered CBF and improved cognitive performance in older adults with and without cognitive impairment [37]. Further research including measurement of CBF is important, to clarify the relationship between CBF, FDG uptake, and Abeta.

However, the majority of the identified studies in a recent review found no significant association between physical activity and the AD biomarkers Abeta 1-42, total tau, and phosphorylated tau in CSF, amyloid PET, hippocampal atrophy on MRI, and parietal temporal hypometabolism with brain FDG [38, 39]. In our analysis, the prognosis for Abeta-positive subjects was significantly different from those who were Abeta negative. The inconsistency between these results may be due to the differing backgrounds of the subjects whose Abeta information was unclear. Further research including the factors Abeta, CGM, and CBF would facilitate clarification of the protective effect of physical activity on AD.

Because Abeta-targeted therapies have been mostly unsuccessful, researchers are becoming increasingly sceptical of the amyloid hypothesis and looking for other potential pathogeneses of AD. It has been observed that Abeta accumulation is associated with tau aggregation, neuroinflammation, and synaptic dysfunction. In this study, elevated Abeta greatly increased the risk of AD in subjects with MCI. Higher values of CGM measured by brain FDG decreased the risk of AD in Abeta-positive subjects, and this protection still remained even in subjects with very highly evaluated Abeta. Atrophy of the hippocampus and entorhinal cortex thickness are also strong risk factors for AD. Our results with brain CGM confirmed that inclusion of these factors would enrich clinical trial design. Approaches designed to increase the physiological protective effect of CGM might be potential primary preventive interventions for AD, along with anti-Abeta agents. Physical exercise or aerobic training still needs further research and data supporting a change in physiological factors, to confirm which exercise and its effect on the prevention of AD.

## Figures and Tables

**Table 1:** Baseline characteristics of subjects with brain PET Abeta.

	MCI		NC	
	Abeta+, *N* = 188	Abeta-, *N* = 236	Abeta+, *N* = 52	Abeta-, *N* = 213
Age, mean (SD)	73.6 (6.7)	70.2 (7.7)^∗^	75.3 (5.8)	72.4 (5.9)^∗^
Sex				
Male	109 (58.0)	123 (52.1)	16 (30.8)	103 (48.4)^∗^
Female	79 (42.0)	113 (47.9)	36 (69.2)	110 (51.6)
MMSE, mean (SD)	27.5 (1.8)	28.5 (1.5)^∗^	29.0 (0.9)	29.1 (1.3)
ApoE				
0	54 (28.7)	159 (67.4)^∗^	25 (48.1)	165 (77.8)^∗^
1	100 (53.2)	67 (28.4)	25 (48.1)	43 (20.3)
2	34 (18.1)	10 (4.2)	2 (3.8)	4 (1.9)
Follow-up time, median (range)	25.87 (4.72-60.89)	32.76 (5.54-60.07)^∗^	19.47 (5.51-47.67)	22.13 (5.05-48.46)

^∗^ *P* < 0.05.

**Table 2:** The risk factors for conversion to AD by Cox regression.

Factors	Univariate	Multivariate
	Hazard ratio (95% CI)	Hazard ratio (95% CI)
Hippocampal volume	0.923 (0.913-0.934)	0.959 (0.933-0.985)
Average-brain FDG	0.867 (0.848-0.887)	0.884 (0.851-0.918)
Cortical thickness of the entorhinal cortex	0.903 (0.887-0.918)	0.941 (0.903-0.981)
Av45	1.468 (1.359-1.585)	1.244 (1.113-1.391)
Sex	0.835 (0.666-1.047)	0.534 (0.324-0.882)
ApoE4 (2)	4.502 (3.191-6.352)	2.063 (0.996-4.276)
(1)	2.892 (2.267-3.690)	2.024 (1.135-3.608)
(0)	Reference	Reference
Intracranial volume	1.082 (1.010-1.159)	
Whole-brain volume	0.997 (0.996-0.998)	
Fusiform volume	0.805 (0.765-0.846)	
Ventricles	1.013(1.009-1.018)	
Age	1.006 (0.990-1.023)	
Education	0.971 (0.934-1.010)	

^∗^ *P* < 0.05. Av45: brain PET amyloid; FDG: CGM based on 18F-FDG-PET.

**Table 3:** Risk classification by brain Abeta and FDG stratified by ApoE.

	Level	No. of events/no. of population	Hazard ratio	95% CI
APOE-*ɛ*4 carrier				
a1. Group by AV45 and FDG	A: AV45≦1.231 and FDG > 5.946	4/103	(reference)	—
	B: AV45≦1.231 and FDG≦5.946	2/18	4.848	0.883-26.627
	C: AV45 > 1.231 and FDG > 5.946	21/89	6.798	2.333-19.808
	D: AV45 > 1.231 and FDG≦5.946	38/68	22.601	8.031-63.605
a2. 1.231 < AV45≦1.462	A: FDG≦5.946	18/35	2.908	2.389-6.089
	B: FDG > 5.946	12/54	(reference)	—
a3. AV45 > 1.462	A: FDG≦5.946	20/33	3.628	1.623–8.106
	B: FDG > 5.946	9/35	(reference)	
APOE-*ɛ*4 noncarrier				
b1. Group by AV45 and FDG	A: AV45≦1.231 and FDG > 5.946	5/272	(reference)	—
	B: AV45≦1.231 and FDG≦5.946	5/39	8.144	2.347-28.253
	C: AV45 > 1.231 and FDG > 5.946	9/58	7.206	2.356-22.039
	D: AV45 > 1.231 and FDG≦5.946	10/18	50.72	17.007-151.257
b2. 1.231 < AV45≦1.462	A: FDG≦5.946	3/10	4.335	0.947-19.84
	B: FDG > 5.946	5/41	(reference)	—
b3. AV45 > 1.462	A: FDG≦5.946	7/8	36945.00	0.00-missing
	B: FDG > 5.946	4/17	(reference)	

*P* < 0.001. AV45: brain PET Abeta; FDG: CGM based on 18F-FDG-PET.

## Data Availability

The data used to support the findings are available from the ADNI database.

## Additional Points

Data used in preparation of this article were obtained from the Alzheimer's Disease Neuroimaging Initiative (ADNI) database (adni.loni.ucla.edu). As such, the investigators within the ADNI contributed to the design and implementation of ADNI and/or provided data but did not participate in the present analyses or the writing of this report. A complete listing of ADNI investigators can be found at http://adni.loni.usc.edu/wp-content/uploads/how_to_apply/ADNI_Acknowledgement_List.pdf.

## Supplementary Materials

Supplementary Materials Supplementary Figure 1: survival tree to find the optimal cut-off point for AV45 and PDG. Supplementary Figure 2: survival curves by AV45 and FDG-PET. Supplementary Table 1: factors associated with conversion to AD stratified by brain PET Abeta.
