# Supplementary material for: Protective Factors Modulate the Risk of Beta Amyloid in Alzheimer's Disease
Source: Behav Neurol. 2020 Oct 29;2020:7029642. doi: 10.1155/2020/7029642 (PMC7647774; doi:10.1155/2020/7029642)

Suppl.Table 1 Factors associated with conversion to AD stratified by brain PET Abeta

	Abeta+<br>Converter<br>N=78	Non-converter<br>N=162	Abeta−<br>Converter<br>N=16	Non-converter<br>N=433
Age, mean (SD)	73.8 (6.4)	74.1 (6.6)	69.6 (7.2)	71.3 (6.9)
Education	16.3 (2.5)	15.9 (2.8)	15.4 (2.8)	16.7 (2.5)*
Sex				
Male	47 (60.3)	78 (48.1)	9 (56.3)	217 (50.1)
Female	31 (39.7)	84 (51.9)	7 (43.7)	216 (49.9)
MMSE, mean (SD)	27.1 (1.8)	28.2 (1.6)*	28.5 (1.9)	28.8 (1.4)
ApoE4#				
0	19 (24.4)	60 (37.0)	10 (72.7)	314 (62.5)
1	45 (57.7)	80 (49.4)	1 (24.5)	106 (25.0)
2	14 (18.0)	22 (13.6)	1 (2.8)	12 (12.5)
Average brain 18F-FDG-PET	5.8 (0.63)	6.3 (0.64)*	6.0 (0.57)	6.6 (0.56)*
Thickness of entorhinal cortex	3237.7 (748.8)	3715.6 (603.9)*	3535.9 (741.3)	3828.2 (637.5)
Hippocampal volume	6341.8 (892.2)	7071.1 (937.0)*	6626.3 (1573.9)	7473.9 (994.4)
Intracranial volume	1549276 (180183)	1500766 (160563)*	1503619 (170279)	1498025 (146538)
Whole brain volume	1049094 (109185)	1045248 (98472.3)	1038108 (101047)	1061237 (102439)
Fusiform gyrus volume	17309.4 (2615.7)	18272.9 (2436.2)*	18269.8 (2070.0)	18871.2 (2376.1)

*p<0.05; #ApoE4 = 0, 1, 2 refers to the number of copies of the ApoE4 allele.

AD, Alzheimer’s disease; MMSE, mini-mental state examination.

Suppl. Figure 1 Survival trees to find the optimal cut-off point for AV45 and PDG

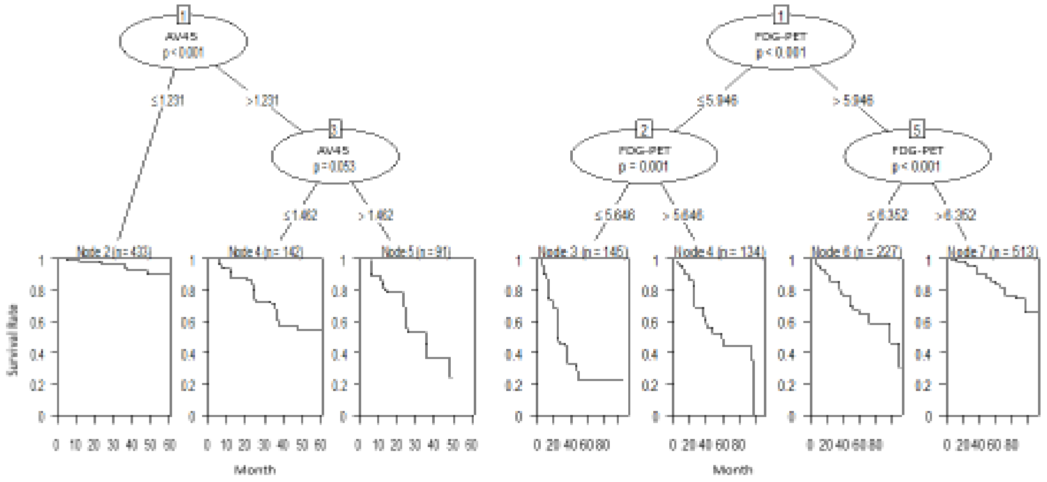

Suppl. Figure 2 Survival curves by AV45 and FDG-PET

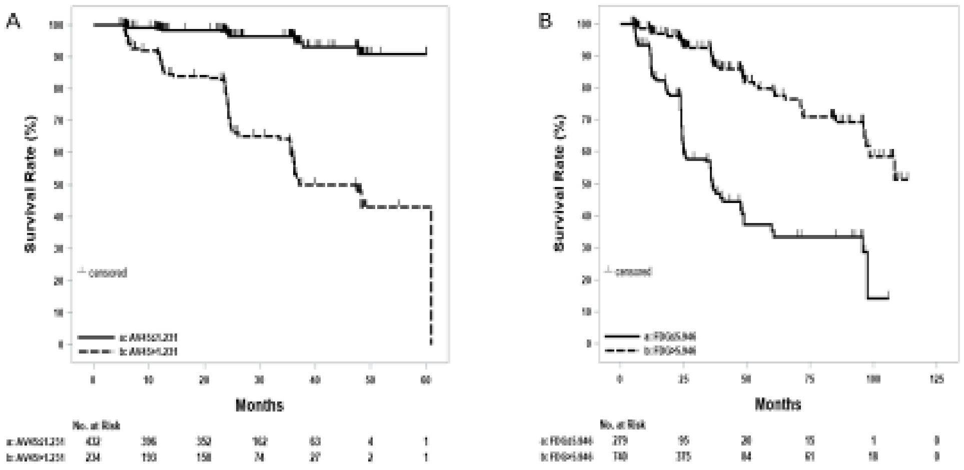

Supplement: Supplementary Materials — Supplementary Figure 1: survival tree to find the optimal cut-off point for AV45 and PDG. Supplementary Figure 2: survival curves by AV45 and FDG-PET. Supplementary Table 1: factors associated with conversion to AD stratified by brain PET Abeta. [file 7029642.f1.pdf]
